# Effects of trophic status on microcystin production and the dominance of cyanobacteria in the phytoplankton assemblage of Mediterranean reservoirs

**DOI:** 10.1038/srep17964

**Published:** 2015-12-09

**Authors:** Maria Antonietta Mariani, Bachisio Mario Padedda, Jan Kaštovský, Paola Buscarinu, Nicola Sechi, Tomasa Virdis, Antonella Lugliè

**Affiliations:** 1 University of Sassari, Dipartimento di Architettura, Design e Urbanistica, Via Piandanna 4, 07100 Sassari, Italy; 2 University of South Bohemia, Faculty of Science, Department of Botany, České Budějovice, Czech Republic; 3 Ente acque della Sardegna, Settore della limnologia degli invasi, Viale Elmas 116, 09122 Cagliari, Italy

## Abstract

The aim of our study was to evaluate the abundance of cyanobacteria and microcystins in four Sardinian reservoirs (Italy) characterised by different trophic status to define a reference picture for future changes. Increasing levels of eutrophication and the abundance of cyanobacteria are expected to occur due to climate change, especially in the southern Mediterranean. Consequently, an in-depth study of the occurrence of harmful cyanobacteria is important to develop appropriate management strategies for water resources at a local scale. Monthly samples were collected at one station in each reservoir over an 18-month period. The Analysis of similarity indicated that cyanobacterial abundance and species composition differed significantly among the reservoirs. The Redundancy analysis highlighted their relationship to trophic, hydrological and seasonal patterns. Spearman’s analysis indicated that there were significant correlations among the most important species (*Planktothrix agardhii–rubescens* group, *Aphanizomenon flos-aquae* and *Dolichospermum planctonicum*), nutrients and microcystins. We highlighted that the species composition during periods of maximum microcystin concentrations differed from those typically reported for other Mediterranean sites. We found new potential microcystin producers (*Aphanizomenon klebahnii*, *Dolichospermum macrosporum* and *Dolichospermum viguieri*), which emphasised the high diversity of cyanobacteria in the Mediterranean area and the need for detailed research at the local scale.

In freshwater environments, several toxic cyanobacterial species form the most common and noxious type of harmful algal blooms (CyanoHABs^1^,^2^), which have potentially serious consequences for human health^3^. As a result of incidents attributed to toxic CyanoHABs, the World Health Organisation (WHO) and several national authorities around the world recommended that risk assessment plans and safety levels include cyanobacteria as a parameter that must be monitored^4^. However, in Italy, there is only a national limit for cyanotoxins and cyanobacteria abundance in bathing water, and no limit has been established for cyanotoxins in drinking water. The provisional WHO guideline of 1 μg l^**−**1^ microcystin-LR is used if local authorities suspect a risk to human health^3^,^5^,^6^. For this reason, it is essential to develop tools for the early prediction of toxic CyanoHABs.

The incidence of blooms has increased worldwide as a result of cultural eutrophication^7^. Consequently, the threats posed by cyanotoxins are greater^1^. However, with the current levels of knowledge, researchers cannot comprehensively evaluate the complex interactions between eutrophication and climate change, though it is likely that climatic factors will result in larger and more frequent bloom events^8^,^9^,^10^,^11^,^12^. Filling these knowledge gaps is particularly important in geographical areas where the natural environmental conditions favour the rapid growth of cyanobacteria and seriously restrict the availability of water. This is the situation at many sites in the Mediterranean basin^13^,^14^,^15^.

Sardinia (Italy) is the second largest island in the Mediterranean Sea. It has only one natural lake, Lake Baratz, and at least 90% of its drinking water is stored in more than 40 reservoirs^16^. Data for Sardinian reservoirs that were collected on a multi-decadal scale show that most were eutrophic, with phytoplankton communities dominated by cyanobacteria^17^,^18^. The presence of cyanotoxins was assessed in a number of Sardinian reservoirs, with microcystins (MCs) being the most frequently detected cyanotoxin^19^,^20^,^21^. These data were derived from short-term surveys that occurred during mono-specific and mixed blooms. The cyanotoxin monitoring program is no longer active in Sardinia, and no research on the relationship between the cyanotoxins and environmental conditions has been conducted.

Our aims were to study the phytoplankton community, the relative abundance of cyanobacteria, the concentrations of MCs and their relationships to environmental variables in Sardinian reservoirs (Italy) and to define a reference picture for future changes. We focused on describing the seasonal dynamics in four reservoirs with different trophic statuses over an 18-month period (from June 2010 to December 2011). We used statistical analyses to test whether the abundances of cyanobacteria and concentrations of MCs were linked to the trophic status and hydrological conditions of the four reservoirs. It is important to understand the relationships between trophic status and cyanobacterial populations^11^,^22^,^23^ and their associated production of MCs in order to optimise the environmental management of reservoirs in the Mediterranean region.

## Materials and Methods

### Study sites

**Figure 1:**
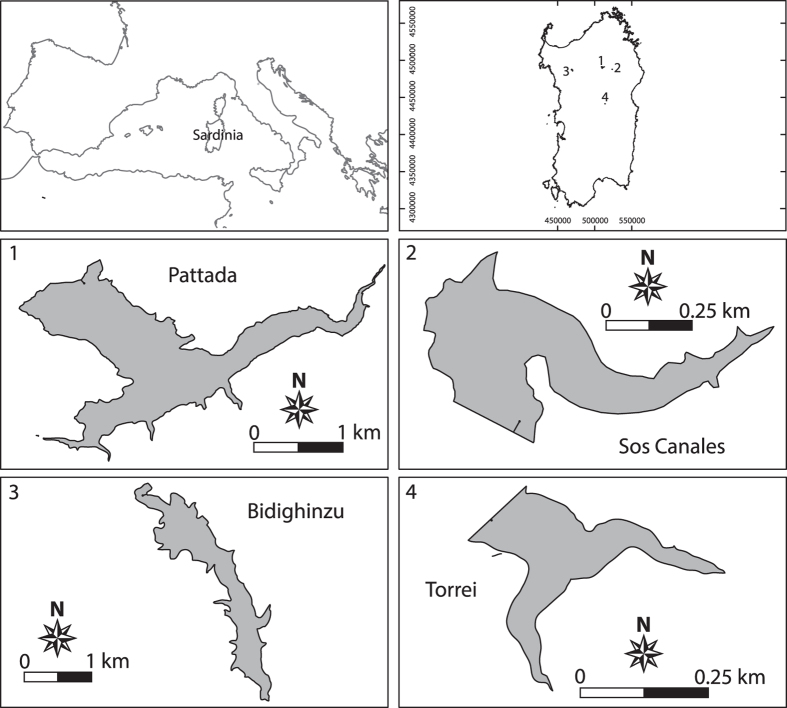
Locations of Sardinian reservoirs and sampling stations. The maps were created and assembled using QGIS software version 2.8.2.

Data were collected from the Bidighinzu (BID), Pattada (PAT), Sos Canales (SCN) and Torrei (TOR) reservoirs (Table 1, Fig. 1), which are located in northern Sardinia (northwest Mediterranean). The four reservoirs are managed by the Regional Agency Ente Acque della Sardegna (ENAS). The main use of the reservoirs is to store drinking water. Only PAT is also used for irrigation. The BID, PAT, and SCN reservoirs belong to the Italian network of long-term ecological research^24^,^25^ (LTER-Italy; www.lteritalia.it).

### Sampling

Sampling was conducted monthly from June 2010 to December 2011 at a single station close to the deepest part of each reservoir. Water samples for environmental and nutrient variables were collected at fixed depths along a vertical profile from the surface (0.5 m), at depths of 1, 2.5, 5, 7.5, 10, 15 and 20 m, and below the last depth at intervals of 10 m to the bottom using Niskin bottles. For phytoplankton, samples were taken from the first six depths listed above (0.5,1, 2.5, 5, 7.5 and 10 m). For the quantitative toxin analysis, 1 l water samples were collected from the surface (0.5 m).

### Environmental variables and phytoplankton

Monthly rainfall values, air temperature and wind intensity, as recorded by the ENAS’s meteo-climatic station located close to the dams, were considered during the period from January 2010 to December 2011 to further characterise the reservoirs. The data for TOR were derived from Sardegna Clima Onlus data network (http://www.sardegna-clima.it), considering the meteo-climatic station closest to the reservoir (Tonara).

Transparency was assessed based on Secchi disk (SD) measurements. Temperature (T), conductivity (CD), pH, and dissolved oxygen (DO) were measured in the field using a multi-parametric probe (Hydrolab Datasonde 5). The values recorded in the field were replicated in the laboratory for pH (Orion Research Model 960, Beverly, MA, USA) and conductivity (Analytical Control 120).

Water samples were stored in cold dark conditions prior to laboratory analyses. We measured alkalinity (Alk), ammonium (NH_4_–N), nitrite (NO_2_–N), nitrate (NO_3_–N), total nitrogen (TN), reactive silica (RSi), orthophosphate (RP), and total phosphorus (TP) according to Strickland and Parsons^26^. Chlorophyll *a* (Chl *a*) was measured as described by Goltermann *et al*.^27^.

Phytoplankton samples (100 ml) were fixed in the field using Lugol’s solution and analysed using Utermöhl’s technique^28^. Cell abundance was determined microscopically from subsamples (5–10 ml) of the fixed samples, using an inverted microscope (Zeiss, Axiovert 10, Oberkochen, Germany) at 200 × and 400 × magnifications, based on cell counts from an appropriate number of fields. Species were identified from samples of live and fixed cells according to the taxonomic guides listed by Marchetto *et al*.^18^, Komárek and Komárková^29^, Komárek and Zapomělová^30^,^31^, and Suda *et al*.^32^. The taxon bio-volume was determined by multiplying the cell abundance of each taxon by the cell volume, which was obtained by geometrical approximations from the measurement of at least 30 specimens, following the method of Sun and Liu^33^. The bio-volume was converted to biomass, based on the assumption that 1 mm^3^ = 1 mg fresh weight biomass^34^.

The environmental and nutrients data were averaged for the entire water column. The Chl *a* and phytoplankton data were averaged for the euphotic zone (Zeu = 2.5 times the Secchi disk depth^35^). The Zeu/Zmix ratio (where Zmix is the mixing depth) was used as a measure of light availability in the mixed layer^36^. The relative water column stability (RWCS) was calculated following Naselli-Flores^37^.

Following Reynolds^38^, we related the dominant and co-dominant phytoplankton species (in terms of biomass) to C–S–R (competitor, C; stress-tolerant, S; ruderal, R) life strategies^38^,^39^ to interpret the phytoplankton responses, in terms of growth strategies, to various environmental conditions at both temporal (seasonal) and spatial (site) scales.

The trophic status of the four reservoirs was assessed based on Vollenweider–OECD classification criteria^40^.

### Toxin determination

MCs and nodularins were detected in water samples by an enzyme-linked immunosorbent assay (ELISA), following Fischer *et al*.^41^. After a freeze-thaw cycle and ultrasonic treatment (ELMA S 10, Elmasonic), the samples were analysed using an ELISA kit (Microcystins ADDA-ELISA Microtiter Plate Abraxis LLC, Warminster, PA), according to the manufacturer’s protocol. Absorbance was read at a single wave length of 450 nm using a Miniphotometer (model 6 +, Metertech Inc.). The results were expressed as MC-LR equivalents, as indicated by the manufacturer, and were reported in the text as MCs. Samples were considered positive when the MCs concentration was higher than the lowest detection limit (0.10 μg l^–1^).

### Data analyses

Non-metric multidimensional scaling analyses (nMDS) of species bio-volumes were conducted to assess the differences among sites. A similarity matrix was obtained using a dataset comprising all the cyanobacterial species. The similarity matrices were based on the Bray–Curtis similarity index. The significance of spatial differences was assessed using a one-way analysis of similarities test (ANOSIM)^42^, and probability percentages of less than 3% were considered significant. The major species contributing to the spatial differences among reservoirs and the similarity among samples at each reservoir were investigated using a similarity percentage analysis (SIMPER)^42^. This analysis broke down the percentage contribution of each species, allowing identification of the species that were most important in creating the observed pattern of difference. A data matrix was constructed considering all the cyanobacterial species abundances in all the samples for each reservoir. The Bray-Curtis similarity measure was implicit to SIMPER.

Ordinations were performed using CANOCO^43^. A detrended correspondence analysis (DCA) was first conducted on potentially toxic cyanobacterial species (selected via SIMPER) and MCs concentrations and indicated a linear distribution (with a gradient length between 2 and 3), which validated the use of a direct linear methodology, such as a redundancy analysis (RDA). For the ordination analysis, the data for abiotic and biological variables were transformed by log_10_ (x + 1). RDA was used to examine the relationships between the abiotic variables (T, CD, Zeu/Zmix, RWCS, NH_4_–N, NO_2_–N, NO_3_–N, TN, RP and TP) and potentially toxic cyanobacterial species and to select the variables that best described the seasonal distribution of potentially toxic cyanobacterial species. The significance of environmental variables in explaining the variance of potentially toxic cyanobacterial species in the RDA was tested using Monte Carlo simulations with 499 permutations.

Non-parametric correlation (Spearman) analyses were used to test the relationships between species selected via SIMPER and environmental variables (SD, T, pH, CD, Alk, DO, Zeu/Zmix and RWCS), nutrients (NH_4_–N, NO_2_–N, NO_3_–N, TN, RP and TP), and Chl *a* and MCs concentrations. For these analyses, we used the data for the cell abundance of potentially toxic cyanobacterial species at 0.5 m, which was the same depth used for the MCs analyses. A Spearman analysis was conducted using XLSTAT 10.10.

## Results

### Environmental conditions and nutrients

**Figure 2:**
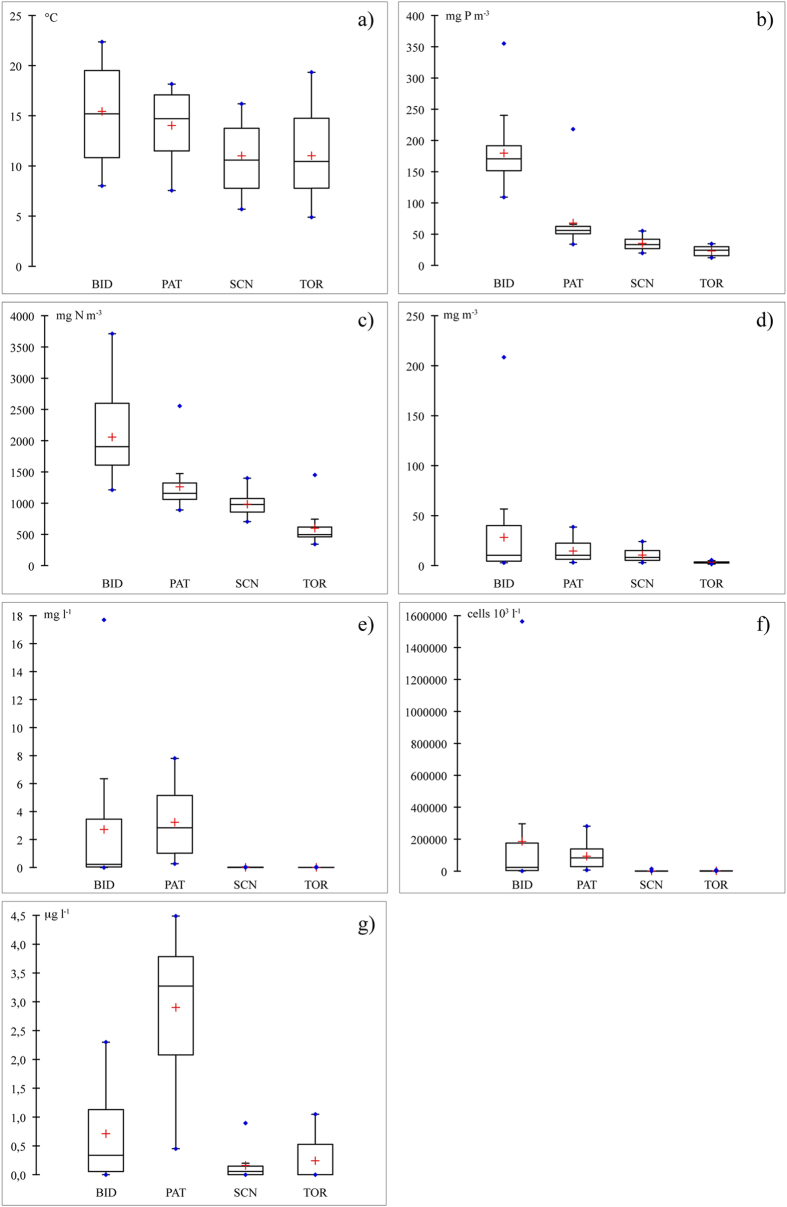
Boxplots of (a) temperature, (b) TP, (c) TN, (d) Chl *a*, (e) biomass, and (f) cell abundance of cyanobacteria and (g) MCs in the reservoirs. Box plots were constructed considering total dataset (all samplings) of averaged values along vertical profile for temperature, TP, and TN; weighted means in the photic zone for Chl *a*, and biomass and cell abundance of cyanobacteria; and MCs concentrations at superficial depth (0.5 m). Line inside box represents the median; cross inside box represents mean; points represent maxima and minima. Bottom of box is the first quartile; upper part is the third quartile. Whiskers represent limits over which values were considered anomalous.

During the study period, the four reservoirs were characterised by different environmental conditions and nutrient concentrations (Table 2). TP and TN clearly increased from TOR to SCN, PAT and BID (Fig. 2). Similarly, there were gradients among the reservoirs for SD transparency, water temperature and Zeu values (Table 2, Fig. 2). There was a stable thermal stratification from June to October in PAT (Zmix: 9.4–10.0 m; Zmax: 40 m) and from June to September in the other reservoirs (BID, Zmix: 5.6–5.8 m, Zmax: 20 m; SCN, Zmix: 5.0–5.8 m, Zmax: 30 m; and TOR, Zmix: 4.4–6.7 m, Zmax: 30 m). Accordingly, RWCS reached its minimum in winter (1 in TOR in November and December, 2 in SCN in December and February, 3 in PAT in December and 4 in BID in February) and its maximum in the summer months (321 in PAT in August, 325 in TOR in July, 346 in SCN in July and 392 in BID in July) (Table 2). The Zeu/Zmix ratio was lowest in the winter-spring months (minimum of 0.03 in PAT in April, 0.04 in BID in December, 0.08 in SCN in February and 0.21 in TOR in April) and highest in summer (maximum of 1.4 in BID in July, 1.5 in PAT in July, 2.5 in SCN in August and 3.0 in TOR in June).

**Figure 3:**
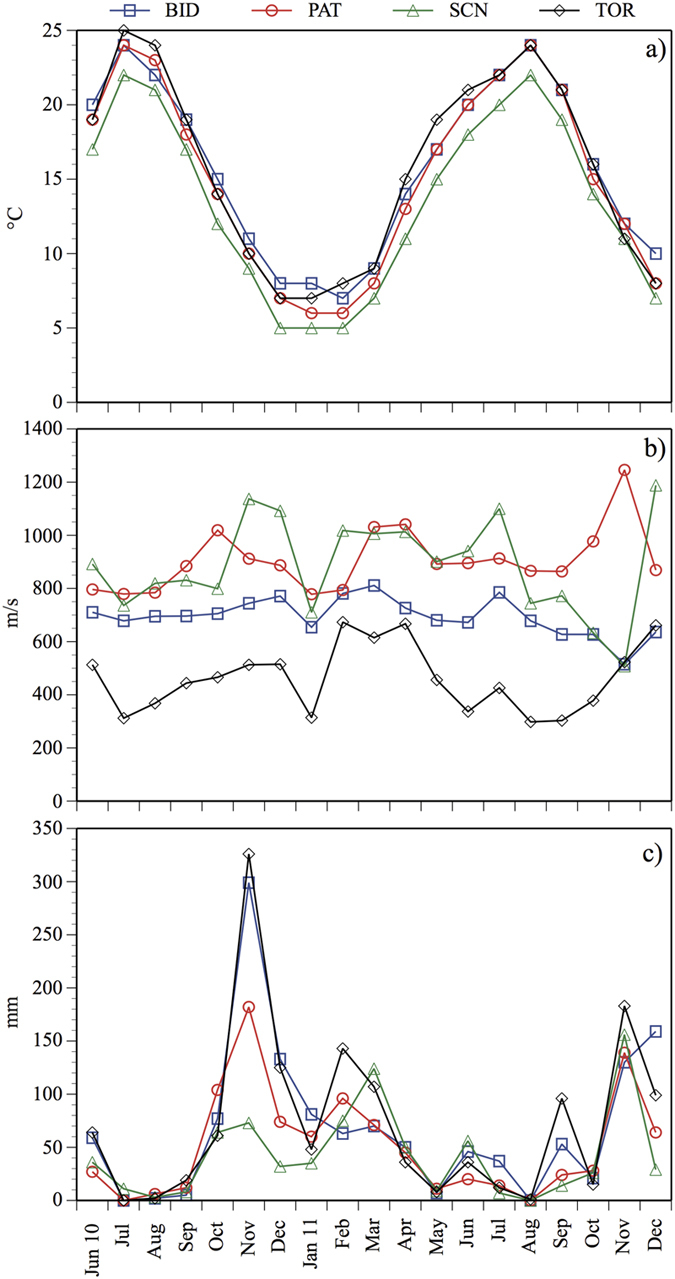
Air temperature (a), wind speed (b) and rainfall (c) dynamics in the four reservoirs during the years 2010 and 2011.

The air temperature increased from SCN to TOR, PAT and BID. The wind intensity increased from TOR to BID, PAT and SCN, and rainfall increased from SCN to PAT, BID and TOR (Table 1). Wind intensity was less variable in BID than in the other reservoirs. Rainfall showed a very similar seasonal pattern in all of the reservoirs, with the maximum in October-December and the minimum in May and August (Table 1, Fig. 3).

According to the Vollenweider–OECD classification criteria^40^, the previously assessed trophic status were confirmed for all the reservoirs (Table 1).

### Phytoplankton

**Figure 4:**
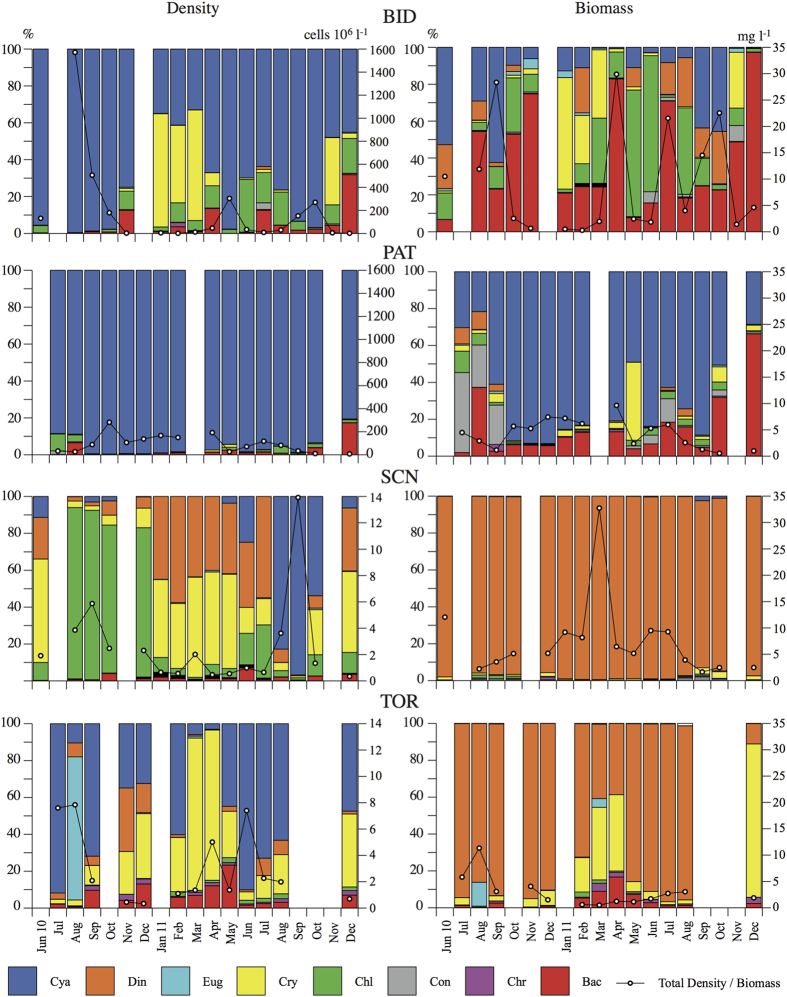
Phytoplankton class composition in terms of cell abundance and biomass with respect to total cell abundance and biomass in Bidighinzu Lake (BID), Pattada Lake (PAT), Sos Canales Lake (SCN) and Torrei Lake (TOR) during the study period. (Bac = Bacillariophyceae; Chr = Chrysophyceae; Con = Conjugatophyceae; Chl = Chlorophyceae; Cry = Cryptophyceae; Eug = Euglenophyceae; Din = Dinophyceae; Cya = Cyanobacteria).

Chl *a*, cell abundance and biomass values increased from TOR to SCN, PAT and BID (Table 2, Fig. 4). The maximum phytoplankton cell abundances occurred in summer–autumn in all of the reservoirs. The maximum biomass values were in spring and autumn in BID, in spring in PAT and SCN, and in summer in TOR (Fig. 4). Cyanobacteria dominated the cell phytoplankton abundance in the hypereutrophic BID (dominant species: *Aphanocapsa* sp. and *Aphanizomenon flos-aquae* (L.)), particularly in the eutrophic PAT (dominant species: *Planktothrix agardhii–rubescens* group). Cyanobacteria also dominated the biomass in PAT, whereas Bacillariophyceae dominated the biomass in BID (dominant species: *Aulacoseira granulata* (Ehrenberg) Simonsen), *Cyclotella ocellata* (Pantocsek) and *Stephanodiscus* sp. (Ehrenberg)) (Fig. 4). Cyanobacteria were less abundant and important, in terms of the percentage contribution to total phytoplankton, in TOR and SCN (Figs 2 and 4). In SCN and TOR, the phytoplankton biomass was dominated by Dinophyceae (dominant species: *Gymnodinium uberrimum* (G.J.Allman) Kofoid and Swezy) and by Cryptophyceae in TOR (dominant species: *Plagioselmis lacustris* (Pascher and Ruttner) P. Javornický) (Fig. 4).

S-strategists (i.e., stress tolerant species), which prefer environments with low nutrient concentrations but high energy conditions, were dominant during stratification periods (high Zeu/Zmix) and prevailed in the reservoirs that had lower trophic status (SCN and TOR; e.g., *Gymnodinium*). R-strategist species (i.e., ruderal species), which are adapted to maximising suspension opportunities and low irradiance values, characterised the periods with low Zeu/Zmix values (mixing periods) in PAT (e.g., *Planktothrix* spp.) and those with high Zeu/Zmix conditions (stratification periods) in BID (e.g., *Aulacoseira*), preferring reservoirs that had higher trophic status. C-strategists (i.e., competitive species), which are adapted to environments with low disturbance levels and saturated by light and nutrients, were particularly present during mixing periods in both hypereutrophic (BID; e.g., *Cyclotella* and *Stephanodiscus* species) and mesotrophic conditions (TOR; e.g., *Plagioselmis lacustris*). Among the cyanobacteria (Table 3), S-strategist and C-strategist species were the most numerous, especially under hypereutrophic and eutrophic status.

### Cyanobacterial species composition and microcystins

**Figure 5:**
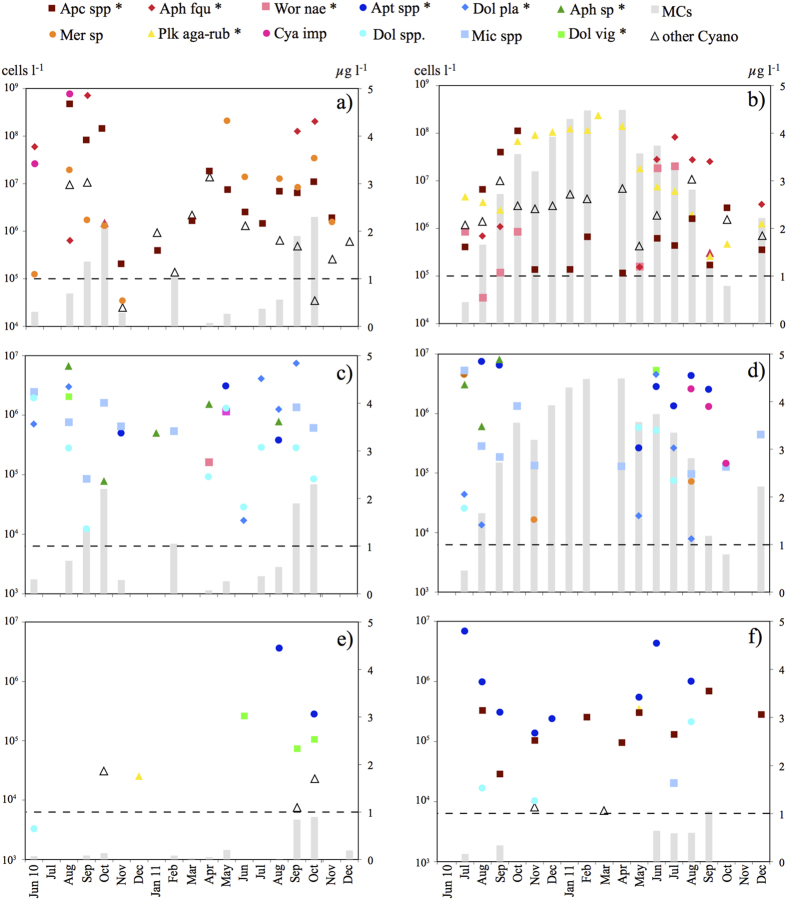
Abundance of dominant cyanobacterial species, and MCs concentrations. (**a**) Species with abundance always >10^4^ cells l^**−**1^ in Bidighinzu Lake; (**b**) species with abundance always > 10^4^ cells l^**−**1^ in Pattada Lake; (**c**) species with abundance always <10^7^ cells l^**−**1^ in Bidighinzu Lake; (**d**) species with abundance always <10^7^ cells l^**−**1^ in Pattada Lake; (**e**) species in Sos Canales Lake and (**f**) Torrei Lake. (* = potentially toxic species; Apc spp* = (*Aphanocapsa* sp.* + Aphanocapsa incerta* + Aphanocapsa delicatissima*); Ant spp* = (*Anathece* sp.* + Anathece chlatrata* + *Anathece minutissima*); Cya imp* = *Cyanocatena imperfecta*; Mer sp = *Merismopedia* sp.; Mic spp* = (*Microcystis* sp.* + Microcystis aeruginosa* + Microcystis flos-aquae* + Microcystis wesembergii*); Wor nae* = *Woronichinia naegeliana*; Dol pla* = *Dolichospermum planctonicum*; Dol vig* = *Dolichospermum viguieri*; Dol spp* = (*Anabaena* sp.* + Dolichospermum macrosporum* + Dolichospermum spiroides* + Dolichospermum flos-aquae*); Aph fqu* = *Aphanizomenon flos-aquae*; Aph sp* = *Aphanizomenon* sp.; Plk agr* = *Planktothrix agardhii-rubescens* group; other Cyano = other cyanobacteria).

Cyanobacterial species were more numerous in BID (32) and PAT (31) than in SCN (10) and TOR (8) (Table 3). The three dominant species, in terms of cell abundance, in hierarchical order were *Aph. flos-aquae*, *Cyanocatena imperfecta* (Cronberg & Weibull) Joosten and *Aphanocapsa* spp. in BID; *Planktothrix agardhii-rubescens* group, *Aphanocapsa* spp. and *Aph. flos-aquae* in PAT; *Anathece* sp., *Dolichospermum viguieri* (Denis and Frémy) Wacklin, Hoffmann and Komárek (=*Anabaena viguieri* Denis and Fremy) and *Planktothrix agardhii-rubescens* group in SCN; and *Anathece* sp., *Aphanocapsa* spp. and *Dolichospermum* spp. in TOR (Fig. 5).

**Figure 6:**
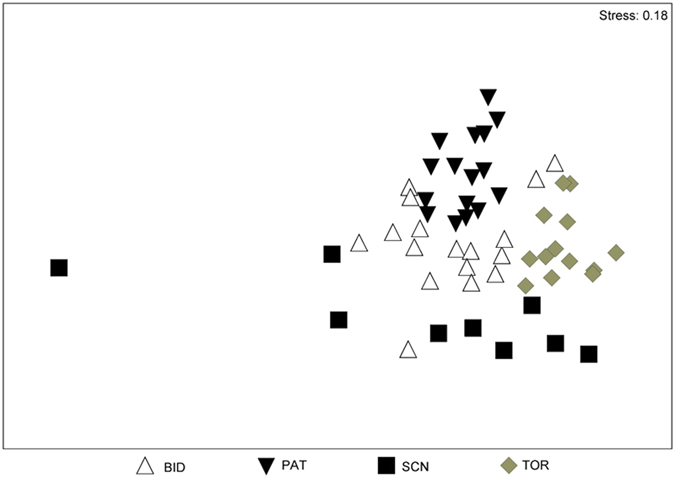
nMDS of differences among sites with respect to biomass of all cyanobacterial species.

The ANOSIM revealed significant differences in the cyanobacterial assemblages among the four reservoirs (ANOSIM Global *R* = 0.55, *p* < 0.001). The nMDS analyses showed that the data clusters were more compact for PAT and TOR (Fig. 6) and, therefore, that the differences among samplings were minimal in these reservoirs. These findings were supported by the SIMPER results, which indicated that the high level of data similarity in samples from PAT (33.69%) was attributable to the *P. agardhii–rubescens* group (75.57% contribution). The greatest differences in the cyanobacterial assemblages were between PAT and SCN (99.50%), between PAT and TOR (99.35%), and between BID and SCN (99.30%). There were smaller but significant differences between SCN and TOR (97.81%) and between BID and PAT (94.02%). *P. agardhii–rubescens* group, *Aph. flos-aquae*, *Aphanizomenon* sp. and *Dolichospermum planctonicum* (Brunnthaler) Wacklin, Hoffmann and Komárek (=*Anabaena planctonica* Brunnth) were the species that were mainly responsible for the differences in cyanobacterial assemblages (contribution >10%) between PAT and BID and between these two reservoirs and SCN and TOR. The species mainly responsible for the differences in cyanobacterial assemblages (contribution > 10%) between SCN and TOR were *Anathece* sp., *Dolichospermum* sp. (*Anabaena* sp.), *D. viguieri*, and *D. planctonicum*.

**Figure 7:**
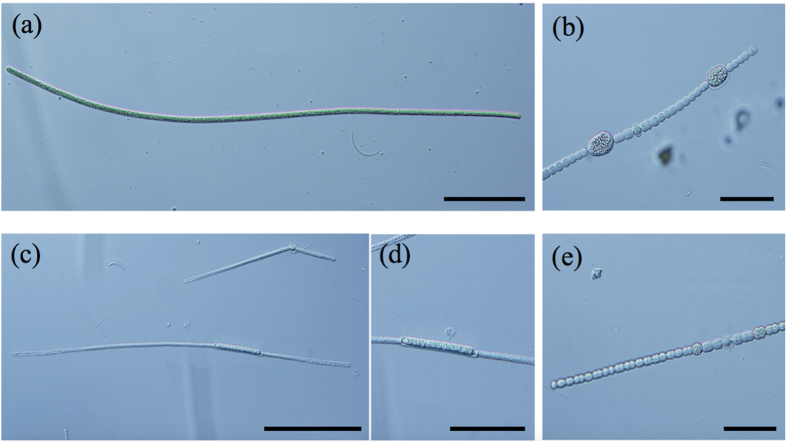
(**a**) *Planktothrix rubescens* group trichome; (**b**) *Dolichospermum macrosporum* and its typical acinetes; (**c**) *Aphanizomenon klebahnii* tricome and (**d**) Its typical acinete; (**e**) *Dolichospermum viguieri* and its typical position of acinetes and heterocysts. (a), (b) and (e) bar=20 μm; (d) bar=50 μm; (c) bar=100 μm.

We detected potentially toxic cyanobacterial species in all of the reservoirs (Table 3). The potentially toxic species identified in this study included the first records for Sardinia for: *Dolichospermum macrosporum* (Klebahn) Wacklin, Hoffmann and Komárek (=*Anabaena macrospora* Klebahn) in BID and PAT; *D. viguieri* in BID, PAT and SCN; and *Aphanizomenon klebahnii* (Elenkin) Pechar and Kalina in BID (Fig. 7).

MCs were detected in 71% of the analysed samples (*n* = 62), including 43% of samples from TOR (*n* = 14), 63% of samples from SCN (*n* = 16), 75% of samples from BID (*n* = 16), and 100% of samples from PAT (*n* = 16). A concentration >1 μg l^–1^ MC-LR was found in almost half of the samples but was found only once in TOR and never in SCN (Fig. 5).

The highest concentrations of MCs were in the summer–autumn period in BID, SCN, and TOR. The highest MC concentrations coincided with high cell abundances at the superficial depth of *Aphanocapsa* sp. and *Aph. flos-aquae* in BID, *D. viguieri* in SCN, and *Dolichospermum* sp. (*Anabaena* sp.) in TOR. In contrast, the maximum MCs concentrations were in PAT in winter–spring; these were the highest concentrations detected in this study. In PAT, the maximum MCs concentrations in winter–spring coincided with the highest cell abundances of cyanobacteria from October to April (from January to April, cell abundance of >181 × 10^6^ cells l^–1^, and MCs concentration of >4 μg l^–1^), due to the *P. agardhii–rubescens* group affirmation. In particular, in samples from December 2010 to April 2011, the dominant species was *P. rubescens* (De Candolle ex Gomont) Anagnostidis and Komárek (=*Oscillatoria rubescens* De Candolle ex Gomont) (Fig. 7).

### Relationships between cyanobacterial species and environmental variables

**Figure 8:**
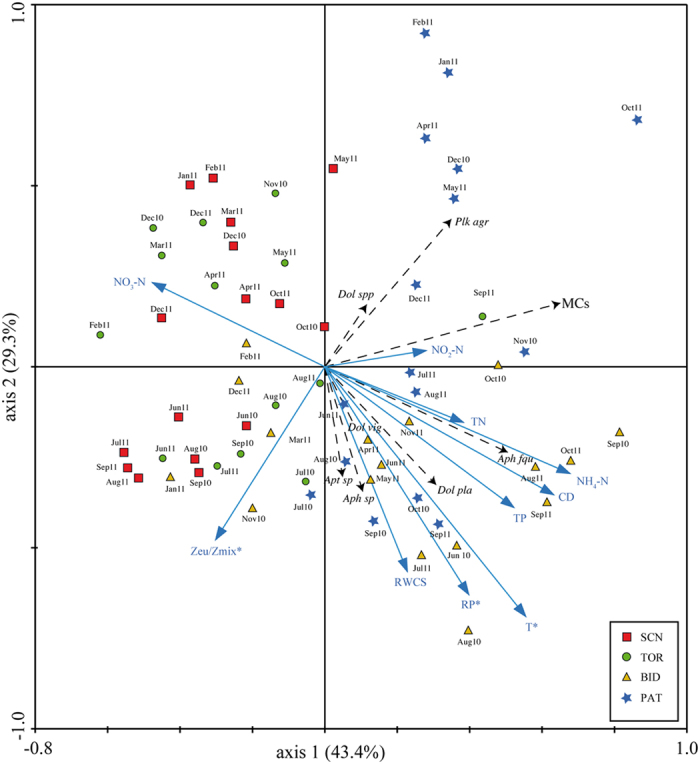
Triplot diagram for RDA of data from four studied reservoirs. Data included environmental variables (explanatory variables), cyanobacterial potentially toxic species (dependent variables), and samples. ***Squares*** Sos Canales Lake samples, ***circles***Torrei Lake samples, ***triangles***Bidighinzu Lake samples, ***stars*** Pattada Lake samples, ***T*** temperature, ***NO***_***3***_^***–***^***N***nitrate, ***NO***_***2***_^***–***^***N*** nitrite, ***NH***_***4***_^***–***^***N*** ammonium, ***TN*** total nitrogen, ***RP*** orthophosphate, ***TP*** total phosphorus, ***Mn*** manganese, ***Fe*** iron, ***SD*** Secchi disk transparency, ***DO*** dissolved oxygen, ***pH***, ***MCs*** microcystins, ***Plk agr** Planktothrix agardhii-rubescens* group, ***Aph fqu** Aphanizomenon flos-aquae*, ***Aph sp.** Aphanizomenon* sp., ***Dol pla** Dolichospermum planctonicum*, ***Dol vig** Dolichospermum viguieri*, ***Dol spp** Dolichospermum* spp., ***Apt spp** Anathece* spp.

The first two axes of the RDA accounted for 72.7% of the variance represented in a cyanobacterial potentially toxic species–environmental variables triplot (axis 1: 43.4%; axis 2: 29.3%; Fig. 8). The RDA indicated that there were three significant variables: T (*F* = 4.71, *P* = 0.002), Zeu/Zmix (*F* = 3.56, *P* = 0.006) and RP (*F* = 2.62, *P* = 0.012). Together, these variables accounted for 16% of the total variance in the cyanobacterial data (25%). The first RDA axis clearly showed the trophic gradient, from reservoirs with lower trophic status (SCN and TOR) on the left, to those with higher trophic status (BID and PAT) on the right. According to the trophic gradient, all of the potentially toxic cyanobacterial species and MCs were positioned on the right. The first axis was positively correlated with NH_4_–N (0.72), T (0.67), and TP (0.65), and negatively correlated with NO_3_–N (−0.24) and Zeu/Zmix (−0.31). The second RDA axis clearly showed, from bottom to top, a seasonal gradient from warmer (summer and autumn) to colder (winter and spring) months. The second axis was positively correlated with NO_2_–N (0.55) and negatively correlated with RWCS (−0.53). The potentially toxic cyanobacterial species were positioned on the negative side of the second axis, consistent with their strong seasonal pattern (summer–autumn). The only exceptions were *Dolichospermum* spp. and, especially, the *P. agardhii–rubescens* group, which were positioned on the positive side of the axis.

Spearman’s analysis indicated that the occurrence of the *P. agardhii–rubescens* group was significantly negatively correlated with transparency (SD; *r* = –0.280, *p* < 0.05) and with RWCS (*r* = –0.272, *p* < 0.05). The occurrence of *Aph. flos-aquae* and *D. planctonicum* were significantly correlated with T (*r* = 0.367, *p* < 0.001 and *r* = 0.407, *p* < 0.001, respectively), RP (*r* = 0.314, *p* < 0.05 and *r* = 0.425, *p* < 0.01, respectively) and TP (*r* = 0.339, *p* < 0.01 and *r* = 0.377, *p* < 0.01, respectively). *Aph. flos-aquae* was also significantly correlated with TN (*r* = 0.292, *p* < 0.05) and NH_4_–N (*r* = 0.281, *p* < 0.05). The *P. agardhii–rubescens* group was strongly significantly correlated with MCs (*r* = 0.740, *p* < 0.0001).

## Discussion

### Trophic status, environmental conditions and phytoplankton

Based on the Vollenweider–OECD classification criteria^40^, there was a trophic gradient among the four reservoirs. These data were consistent with the results of previous studies^16^,^17^ and supported our choice of these reservoirs as prototypical examples of reservoirs with different trophic status for inclusion in our study.

Nutrient enrichment of water bodies (eutrophication) is considered the main driver of cyanobacterial abundance^8^,^44^ and persistence^23^,^45^,^46^. Therefore, we expected that the occurrence of cyanobacteria and their toxic blooms would increase along the trophic gradient of the reservoirs. We found that cyanobacterial abundance differed significantly among the reservoirs (ANOSIM results), with greater abundance in the eutrophic and hypereutrophic PAT and BID than in the oligo- and meso-eutrophic SCN and TOR. The RDA clearly highlighted this relationship, and Spearman’s analysis indicated that there were significant correlations among the most abundant species in eutrophic reservoirs (i.e., *P. agardhii–rubescens* group, *Aph. flos-aquae* and *D. planctonicum*), nutrients, and MCs. The RDA also revealed seasonal patterns. These findings were consistent with the ecological preferences of many potentially toxic cyanobacterial species, such as *Aph. flos-aquae* and *D. planctonicum*, which prefer high summer temperatures and high water stability^16^. By contrast, the ecological preferences (low irradiance and low water stability) of the *P. agardhii–rubescens* group agreed with its autumn-winter affirmation in PAT. Although there was a temperature gradient among the four studied reservoirs (Table 2, Fig. 2), their monomictic character was evident from the stable thermal stratification throughout the summer–autumn period. In general, during thermal stratification, the hydrology of Mediterranean reservoirs favours nutrient accumulation in the deeper water layers. The nutrient levels can increase because of nutrient release from sediment under anoxic conditions, and long periods of stratification can increase hypolimnetic oxygen depletion^47^. Operational procedures and water level fluctuations in summer months can cause the breakdown of the thermal stratification and the re-distribution of nutrients during the period that is most favourable for phytoplankton growth^48^. Moreover, the water turbulence generated during the emptying phase, along with the disruption of stratification, can increase the amount of particulate material in the water column because of re-suspension from the bottom^49^. Based on our data, these effects appear to be less pronounced in these Sardinian reservoirs because they are generally deeper and more interconnected, thus ensuring the persistence of stratification until autumn^16^,^18^,^25^. Similar scenarios for other deep Mediterranean reservoirs were reported in other studies. For example, seasonal variations in thermal stratification were maintained during selective water withdrawal^50^ and during refilling of the reservoir via a water pump located on the river^51^ or via the natural flow of a river tributary^36^.

Long periods of stable stratification are competitively advantageous for gas-vacuolated cyanobacterial species (e.g., *Microcystis, Dolichospermum*, and *Aphanizomenon*), which can regulate their position in the water column (buoyancy) to optimise the use of resources (nutrients and light)^8^. These genera were reported to be the main CyanoHAB producers in the Mediterranean in the summer–autumn period^13^. In our study, a similar case was observed in BID and SCN, where *Aphanizomenon* and *Dolichospermum* (*Anabaena*) were important genera. *Aphanizomenon* and *Dolichospermum* (*Anabaena*) are considered S-strategists, preferring high hydrological stability and high Zeu/Zmix^38^,^52^. By contrast, long-lasting stratification with consequent high levels of nutrient enrichment of the hypolimnion, followed by mixing of the entire water column in late autumn, can significantly affect the cyanobacterial species composition, thus favouring, for example, *Planktothrix* blooms^8^. This was the case for PAT, which was subjected to a longer stratification period and the extensive growth of *Planktothrix* species from late autumn to spring during mixing. *Planktothrix* species are considered R-strategists because they are adapted to maximising suspension opportunities and low irradiance values^38^.

### Species composition of Cyanobacteria

The occurrence of more than 20 species of potentially toxic cyanobacteria followed the trophic gradient in PAT and BID, whereas in SCN and TOR there were 10 or fewer species. We identified cyanobacterial species that were previously reported in Sardinian reservoirs^17^,^18^ and from other Mediterranean sites^13^,^52^. However, we also found species that were not previously reported in these areas. These species included *D. macrosporum* (Klebahn) Wacklin, Hoffmann and Komárek (Fig. 4), *Aph. klebahnii* (Elenkin) Pechar and Kalina, and *D. viguieri* (Fig. 4). *D. macrosporum* (found in BID and PAT) was reported in Britain^53^, France^54^, Romania^55^, Germany^56^, Spain^57^ and the Czech Republic^58^, and it is known to be toxic^57^. *Aph. klebahnii* (found in BID) was reportedly distributed in temperate zones^59^, the Baltic Sea^60^, and Lithuania^61^, and it is thought to be potentially harmful because it produces MCs^62^. *D. viguieri* (found in BID, PAT and SCN) was reported in Britain^53^, the Czech Republic^63^, Greece^64^, and Russia^65^, and it is a potential MCs producer^66^.

*Aphanocapsa* sp., *Aph. flos-aquae*, *D. viguieri*, *Dolichospermum* sp. (*Anabaena* sp.), and the *P. agardhii–rubescens* group, especially *P. rubescens*, were the most important in determining the maximum of the cyanobacteria abundance in the studied reservoirs. This result suggested that there was a marked reduction in the occurrence and importance of *Microcystis* compared with previous scenarios in the same reservoirs. *M. aeruginosa* (Kűtzing sensu Teiling) and *M. flos-aquae* (Wittrock) Kirchner were the dominant cyanobacteria in the summer–autumn periods in BID^17^, and *Microcystis* spp. were dominant in the summers of 1988, 1997, and 1999 in PAT^67^. *Planktothrix* spp. dominated the winter–spring months between 2004 and 2005 in TOR^20^ but were rarely found in this study. *Dolichospermum planctonicum* and *D. ellipsoides* (Bolochoncev) (=*Anabaena elliptica* Lemmermann) were reported as dominant in SCN in the summer–autumn months of 1993, following the strong dominance of *Aphanocapsa* and *Anathece* of 1991 and 1992^68^. In the present study, we detected significant quantities of these members of the Chroococcales and detected *D. viguieri* for the first time.

### Cyanobacteria and toxicity

We found a progressive increase in the occurrence of cyanobacterial abundance and MCs in response to increasing trophic status in these four Sardinian reservoirs. Nutrient over enrichment of waters by human pressure, such as wastewaters from urban centres, agricultural, and industrial activities, has promoted the growth of cyanobacteria as harmful algal blooms^10^. In most cases, the delimitation of the factors controlling and promoting the development of cyanotoxins was not clear and was very difficult to address^69^. Focusing on MCs, several studies revealed a huge variability in the concentrations. Salmaso *et al*.^69^ indicated that most of this variability was due to differences in the cyanobacterial biomass in lakes of different trophic status. The authors also highlighted the importance of the fraction of toxic and non-toxic genotypes within cyanobacterial species and physiological variation in toxin quota (MC concentration per cell or unit biomass) in toxic populations. In approximately 50% of the samples analysed in our study, the concentration of MCs exceeded the MC-LR drinking water guideline (>1 μg l^–1^)^70^, which indicated that the waters of the hypereutrophic BID and, especially, the eutrophic PAT posed a significant threat to human health. Surprisingly, the WHO limit was exceeded once in TOR, and MCs were detected on other occasions, including in SCN. Messineo *et al*.^20^ reported that MCs were detected in TOR in 2004 and 2005, corresponding to abundant *Planktothrix*, but the concentrations were always low (<1 μg l^–1^). Overall, the proportion of positive samples was similar to that reported in a number of studies, including those conducted in other countries^13^,^64^. Our data further emphasised the need for the continuous monitoring of cyanobacterial toxicity, especially in areas where resources are limited, such as in Sardinia and the Mediterranean basin in general.

In our study, the maximum MCs concentrations were detected in summer–autumn, when the reservoirs were stratified and the temperature was 20–30 °C, except in PAT. This seasonality was consistent with that observed in other studies on Mediterranean reservoirs^13^,^71^. However, Cook *et al*.^64^ indicated that cyanobacterial blooms may start before summer and persist until after autumn. Cook *et al*.^64^ and Carrasco *et al*.^13^ also reported that *M. aeruginosa* was the most widespread and most frequently occurring toxic species in the entire Mediterranean region, thus confirming the findings of Abdel-Rahman *et al*.^72^ and Oudra *et al*.^73^. A toxic strain of *M. aeruginosa* was isolated from a reservoir in northern Sardinia, Lake Liscia, and was associated with a fish-kill event^19^. In our study, *M. aeruginosa* was never dominant during periods of peak MCs concentrations. Rather, these periods coincided with the occurrence of high numbers of Chroococcales in the genus *Aphanocapsa* (BID); N_2_-fixing Nostocales, including *Aph. flos-aquae* (BID) and *D. viguieri* (SCN) and *Dolichospermum* sp. (*Anabaena* sp.) (TOR); and Oscillatoriales, including members of the *P. agardhii–rubescens* group (PAT). *Aph. flos-aquae* dominated in the more eutrophic reservoirs (BID and PAT). Spearman’s correlation analysis and RDA indicated that the occurrence of *Aph. flos-aquae* was significantly correlated with the main nutrients (RP, TP, NH_4_–N, and TN) and temperature, which was consistent with its preference for summer months. *Aph. flos-aquae* was linked to saxitoxin production in the United States. *Aphanizomenon* spp. was linked to saxitoxin production in Greece^74^, anatoxin-a production in Finland and Germany^75^ and cylindrospermopsin production in Poland^76^ and Germany^77^. However, the production of MCs by *Aphanizomenon* species has not been conclusively demonstrated^71^,^78^. In our case, although the period of peak MCs partly coincided with high cell abundances of *Aph. flos-aquae* in BID and PAT, it was likely that other cyanobacterial species present at the same time (i.e., *Microcystis* spp., *D. planctonicum* and *Aphanocapsa* spp.) were responsible for the high MCs concentrations. In contrast, the toxicity of various *Dolichospermum* (*Anabaena*) species, including *D. vigueri*, was well documented^75^,^79^,^80^. A strain of *D. planctonicum* (=*A. planctonica*) from Mulargia Lake (south-central Sardinia) was shown to produce unidentified MC–like peptides^20^, and this species dominated in Sardinian reservoirs whose waters contained high concentrations of extracellular MCs^20^. Finally, because large populations of *Aphanocapsa* species coincided with high MCs concentrations in BID and PAT, further studies are required to evaluate their toxicity and ecology in the Mediterranean basin. To date, the ability of *Aphanocapsa* to produce MCs was only demonstrated for *Aphanocapsa cumulus*^81^ from Botswana, which is more widely distributed in tropical regions^82^.

The MCs maxima occurred in winter–spring in PAT during a bloom of the *P. agardhii–rubescens* group, which confirmed the occurrence of harmful strains of this group in Mediterranean reservoirs. This result also illustrated that the ecological requirements of these species differed from those of the other species in this study. In general, *Planktothrix* show strong growth in relatively low-nutrient conditions, potentially enabling them to inhabit waters with a wide range of trophic status^69^. Since the mid-1980s, these species have been reported from a number of Mediterranean sites, including natural lakes^83^, reservoirs^20^,^84^,^85^, and lagoons^86^. The abundance of *Planktothrix* increased in the more eutrophic lakes south of the Alps^87^. In Lake Garda, the appearance of mesotrophic and eutrophic species (i.e., *P. rubescens*) after the 1970s and during the 1980s illustrated the strong effect of eutrophication on the phytoplankton community^88^. A similar trend was observed in Lake Maggiore over a long period^89^. Among the species in this group, *P. rubescens* is one of the most widespread toxin-producing cyanobacteria in European lakes^83^,^90^. It is thought to be the species that produces the highest concentration of MCs per cell among all known cyanobacteria^91^. The red-pigmented *P. rubescens* was reported to be the most representative cyanobacterium in the subalpine region, and it is common in deep and well-stratified lakes^92^,^93^. In Sardinia, *P. rubescens* caused the extremely dangerous cyanotoxin events that occurred in the winter months in the Mulargia and Flumendosa Lakes in 1985^20^. By contrast, *P. agardhii* (Gomont) Anagnostidis and Komàrek, another species in the *P. agardhii-rubescens* group, is most frequently observed in shallow and eutrophic lakes and is frequently found in the northern hemisphere^84^. Bonilla *et al*.^94^ reported that *P. agardhii* (Gomont) Anagnostidis and Komárek is common in eutrophic freshwaters, based on analysis of a large database (940 samples) covering different climatic regions and the Northern and Southern hemispheres. Toxic strains of this species were also isolated from several European lakes^95^.

The most abundant cyanobacterium in this study, *P. rubescens*, was reported to produce concentrated metalimnetic populations during thermal stratification in summer and autumn, when sufficient light penetrated into these water layers^93^,^96^. The presence of *P. rubescens* becomes evident when the trichomes move towards the surface during mixing in winter or when the water turbidity increases. The vertical distribution of the *P. agardhii-rubescens* group in PAT was consistent with this pattern (data not shown). In Sardinian reservoirs, transparency minima were linked both to summer phytoplankton growth and winter sediment runoff from the catchment^97^, accentuated by the strong seasonality of rainfall. Consistent with these results, the Spearman’s correlation analysis and RDA in this study indicated that the *P. agardhii–rubescens* group was negatively correlated with Secchi disk transparency. In any case, this relationship may depend, at least partly, on the increased turbidity resulting from the abundance of the *P. agardhii–rubescens* group in the mixing period. Excessive growth of this species produces characteristic reddish water discoloration, which generates alarm among nearby residents and lake managers. Our data suggest that this alarm is warranted because of the high toxin concentrations in PAT during the periods of maximum cell abundance of this species. On these occasions, the WHO MC limit was substantially exceeded for long periods (Fig. 5).

## Conclusions

Our study complemented and updated what was already known for the study areas^16^,^17^,^18^,^20^,^25^,^67^,^68^,^98^,^99^ and enabled us to support the findings of other similar case studies in other geographical areas^97^,^100^,^101^. Our study provided important evidence for the strong relationships among trophic status, cyanobacterial abundance, and MCs concentrations in Sardinian reservoirs, indicating a key role of nutrients in determining cyanobacteria affirmation. Moreover, we confirmed that the temperature and Zeu/Zmix ratio were important factors in cyanobacteria development^10^,^12^,^94^,^102^,^103^. However, the water management of reservoirs, linked to the different needs and uses, further increases the ecosystem complexity and the difficulty in understanding the multiple relationships between biotic and abiotic components.

We also highlighted that the species composition during periods of maximum MCs concentrations in the four reservoirs differed from those typically reported for other Mediterranean sites^13^,^64^ and that the *P. agardhii-rubescens* group was dominant during the period in which the MCs concentrations substantially exceeded the WHO limit. Our results included the first reports of some potentially toxic species in reservoirs of the Mediterranean basin, emphasising the importance of long-term studies for the early detection of community changes. This is especially important when the changes involve harmful species. Phytoplankton responses to nutrient variations cannot be separated from responses to other, larger environmental changes occurring at the same time, such as global climate change. In particular, increasing levels of eutrophication and the abundance of cyanobacteria are expected due to climate change, especially in the southern Mediterranean and islands. Cyanobacteria are also expected to be favoured by higher temperatures^10^,^103^, especially at lower levels of nutrient concentrations^12^. In any case, the progressive enlargement of the geographical distribution of harmful species both in Mediterranean area and globally^104^,^105^,^106^ highlights the need for further detailed research on their ecology, toxicology, and genetics at the local scale.

## Additional Information

**How to cite this article**: Mariani, M. A. *et al*. Effects of trophic status on microcystin production and the dominance of cyanobacteria in the phytoplankton assemblage of Mediterranean reservoirs. *Sci. Rep*. **5**, 17964; doi: 10.1038/srep17964 (2015).

## Figures and Tables

**Table 1:** Physical parameters, trophic status, water uses and meteo-climate data in the 2010–2011 period for the four reservoirs.

Lake	Year	Altitude	Area	Volume	Zmed	Catchment basin	Trophic status	Use	Air temperature	Wind speed	Rainfall
		(m a.s.l.)	(10^6^ m^2^)	(10^6^ m^3^)	(m)	(10^6^ m^2^)			(°C)	(m s^−1^)	(mm)
BID	1958	334	1.7	12.6	7.3	52	IE	AL	15 (7-24)	717 (515-958)	850 (0-299)
PAT	1984	561	4.4	76	14.9	160	E	AL, IR	14 (6-24)	915 (778-1246)	656 (0-182)
SCN	1956	714	0.3	4.34	13.2	16	ME	AL	12 (4-22)	929 (508-1332)	534 (0-156)
TOR	1968	800	0.2	0.96	17.6	14	OM	AL	14 (4-25)	483 (298-766)	915 (0-326)

(Year = year of construction; Zmed = mean depth; Trophic status: IE = hypereutrophic, E = eutrophic, ME = mesotrophic, OM = oligomesotrophic; Use: AL = alimentary, IR = irrigation).

**Table 2:** Mean, standard deviation, and minimum and maximum values of the main parameters in the reservoirs.

Lake	Tem °C	pH	Con μS cm^−1^	Alk meq l^−1^	RP mg m^−3^	TP mg m^−3^	NO_3_-N mg m^−3^	NO_2_-N mg m^−3^	NH_4_-N mg m^−3^	TN mg m^−3^	RSi mg l^−1^	Chl *a* mg m^−3^	SD m	Cya cells 10^6^ l^−1^	Zeu m	Zmix m	Zeu/Zmix	RWCS
**BID**	15.4 ± 23.5	8.02 ± 8.62	451 ± 507	2.24 ± 2.54	90 ± 140	180 ± 263	548 ± 569	15 ± 18	204 ± 561	2057 ± 2364	4.78 ± 4.20	28.3 ± 53.9	0.90 ± 1.18	176 ± 365	2.24 ± 0.83	15.15 ± 8.94	0.31 ± 0.36	142 ± 133
	8.0–22.4	7.40–8.47	387–537	1.74–2.59	53–151	109–355	28–1435	4–48	27–667	1212–3713	0.99–8.15	2.9–208.5	0.30–1.40	0–1564	0.75–3.5	2.5–26	0.04–1.4	4–392
**PAT**	14.0 ± 19.8	7.47 ± 7.75	280 ± 335	0.78 ± 0.93	17 ± 20	68 ± 103	212 ± 170	9 ± 17	163 ± 412	1263 ± 1273	0.85 ± 1.09	14.6 ± 49.7	2.07 ± 4.34	79 ± 88	5.17 ± 2.36	24.38 ± 17.72	0.47 ± 0.46	139 ± 111
	7.6–18.2	6.71–8.08	249–331	0.62–1.05	0–66	34–218	21–367	1–17	18–624	891–2556	0.27–1.89	3.1–38.6	0.60–3.50	0–281	1.5–8.75	5–47	0.03–1.5	3–321
**SCN**	11.0 ± 13.9	6.94 ± 7.03	163 ± 191	0.38 ± 0.42	4 ± 6	35 ± 39	308 ± 389	4 ± 4	24 ± 30	984 ± 1067	5.62 ± 5.65	10.6 ± 17.9	3.08 ± 3.95	1 ± 4	7.70 ± 3.12	20.44 ± 14.08	0.94 ± 0.91	151 ± 125
	5.7–16.2	6.48–7.28	133–196	0.31–0.46	2–7	20–55	160–576	3–6	15–37	705–1399	5.37–5.96	3.0–24.1	1.15–6.15	0–13	2.87–15.37	5–37	0.08–2.5	2–346
**TOR**	11.0 ± 16.1	7.27 ± 7.47	168 ± 203	0.56 ± 0.59	2 ± 3	23 ± 35	161 ± 154	3 ± 4	37 ± 75	594 ± 785	3.08 ± 3.13	3.4 ± 4.1	3.58 ± 1.26	1 ± 2	8.94 ± 3.14	13.93 ± 10.22	1.03 ± 0.97	148 ± 129
	4.9–19.3	6.83–7.84	141–207	0.38–0.82	1–5	12–35	14–576	1–8	11–233	343–1451	2.91–3.55	1.6–5.6	1.70–6.00	0–7	4.25–15	2.5–28	0.21–3	1–325

(Tem=Temperature; Con=Conductivity; Alk=Alkalinity; RP = Reactive Phosphorus; TP = Total Phosphorus; NO_3_-N = Nitrate; NO_2_-N = Nitrite; NH_4_-N = Ammonium; TN = Total Nitrogen; RSi = Reactive Silica; Chl *a* = Chlorophyll *a*; SD=Secchi disk; Cya=Cyanobacteria; Zeu = Euphotic depht; Zmix = Mixing depth; RWCS = Relative Water Column Stability).

**Table 3:**
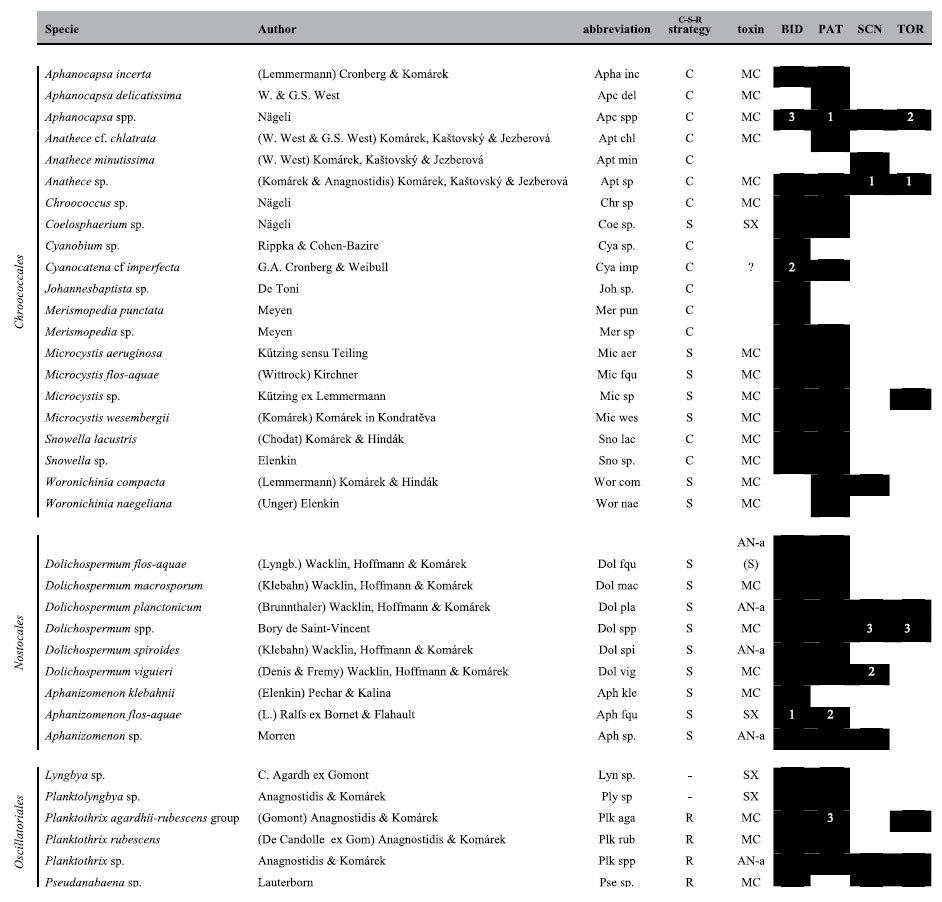
Cyanobacterial species and their presence (black) in the four reservoirs: Bidighinzu (BID), Pattada (PAT), Sos Canales (SCN) and Torrei (TOR).

(C-S-R strategy = competitor, C; stress tolerant, S; ruderal, R). (MC = Microcystin; SX = Saxitoxin; AN-a = Anatoxin-a; AN-a (S) = Anatoxin-a (S)). Numbers indicate the dominant species in hierarchical order during summer months.
